# Feasibility of *BRCA1/2* Testing of Formalin-Fixed and Paraffin-Embedded Pancreatic Tumor Samples: A Consecutive Clinical Series

**DOI:** 10.3390/diagnostics11061046

**Published:** 2021-06-07

**Authors:** Rossella Bruno, Elisa Sensi, Cristiana Lupi, Mirella Giordano, Laura Bernardini, Caterina Vivaldi, Lorenzo Fornaro, Enrico Vasile, Daniela Campani, Gabriella Fontanini

**Affiliations:** 1 Unit of Pathological Anatomy, University Hospital of Pisa, 56126 Pisa, Italy; rossella.bruno@for.unipi.it (R.B.); elisasensi@virgilio.it (E.S.); cristianalupi@libero.it (C.L.); 2 Department of Translational Research and of New Surgical and Medical Technologies, University of Pisa, 56126 Pisa, Italy; mirella.giordano@for.unipi.it (M.G.); laura.bernardini94@gmail.com (L.B.); caterinavivaldi@gmail.com (C.V.); 3 Unit of Medical Oncology, University Hospital of Pisa, 56126 Pisa, Italy; lorenzo.fornaro@gmail.com (L.F.); envasile@gmail.com (E.V.); 4 Department of Surgical, Medical and Molecular Pathology and Critical Area, University of Pisa, 56126 Pisa, Italy; daniela.campani@med.unipi.it

**Keywords:** pancreatic cancer, next generation sequencing, *BRCA1/2*, PARP inhibitors

## Abstract

Pancreatic ductal adenocarcinoma (PDAC) is an aggressive cancer, with most patients diagnosed at advanced stages. First-line treatment based on a combined chemotherapy (FOLFIRINOX or gemcitabine plus nab-paclitaxel) provides limited benefits. Olaparib, a PARP inhibitor, has been approved as maintenance for PDAC patients harboring germline *BRCA1*/2 pathogenic mutations and previously treated with a platinum-based chemotherapy. *BRCA1/2* germline testing is recommended, but also somatic mutations could predict responses to PARP inhibitors. Analysis of tumor tissues can detect both germline and somatic mutations and potential resistance alterations. Few data are available about *BRCA1/2* testing on pancreatic tumor tissues, which often include limited biological material. We performed *BRCA1/2* testing, by an amplicon-based Next Generation Sequencing (NGS) panel, on 37 consecutive PDAC clinical samples: 86.5% of cases were adequate for NGS analysis, with a success rate of 81.2% (median DNA input: 10 nanograms). Three *BRCA2* mutations were detected (11.5%). Failed samples were all from tissue macrosections, which had higher fragmented DNA than standard sections, biopsies and fine-needle aspirations, likely due to fixation procedures. *BRCA1/2* testing on pancreatic tumor tissues can also be feasible on small biopsies, but more cases must be analyzed to define its role and value in the PDAC diagnostic algorithm.

## 1. Introduction

Pancreatic cancer is one of the main leading causes of cancer death worldwide [1] and its incidence is increasing both in the United States and in Europe [2]. Risk factors associated with this tumor include obesity, diabetes and tobacco use, with about 10% of cases having a genetic cause [3]. In fact, different cancer syndromes are associated with an increased risk of developing pancreatic cancer, such as the Peutz-Jeghers syndrome, due to mutations in the serine/threonine kinase 11 *(STK11)* gene, and the hereditary breast-ovarian cancer syndrome due to mutations in BRCA1 DNA repair associated *(BRCA1)* and BRCA2 DNA repair associated *(BRCA2)* genes. In particular, mutations in *BRCA2* are among the most common inherited risk factors (5–17% of familial cases) [3,4,5].

The majority of pancreatic cancers are malignancies of the exocrine pancreas, and ductal pancreatic adenocarcinoma (PDAC) is the most common histotype [3,6]. PDAC usually arises from precursor intraepithelial neoplasias and in a minority of cases from intraductal papillary mucinous neoplasms [7]. PDAC is challenging to treat with a 5-year survival below 9% [8,9]; indeed, initial symptoms are vague and unspecific, and only 15–20% of patients are eligible for surgery, with most of them experiencing recurrence within 5 years [9].

Systemic chemotherapy combinations with oxaliplatin, irinotecan, fluorouracil and leucovorin (FOLFIRINOX) or gemcitabine plus nab-paclitaxel are the standards of care for locally advanced and metastatic disease, but they offer only a limited effectiveness [3,9].

Comprehensive genomic analyses have identified commonly mutated oncogenes and onco-suppressors in pancreatic cancers, such as KRAS proto-oncogene, GTPase *(KRAS)*, cyclin dependent kinase inhibitor 2A *(CDKN2A)*, tumor protein p53 *(TP53)* and SMAD family member 4 *(SMAD4)*, but none of them is clinically targetable with currently approved therapeutic regimens. The high genomic heterogeneity of PDAC can explain the relatively slow progress in the development and approval of effective targeted therapies [10].

The latest American Society of Clinical Oncology (ASCO) guidelines recommended the early testing of actionable genomic alterations for PDAC patients to evaluate additional treatment after first-line therapy. In detail, PDAC should be tested for microsatellite instability, *BRCA1* and *BRCA2* mutations and neurotrophic receptor tyrosine kinase (*NTRK*) gene fusions in order to consider treatment with immune check-point inhibitors, poly(ADP-ribose) polymerase 1 (PARP) inhibitors and TRK inhibitors, respectively [11]. 

In particular, PARP inhibitors have recently emerged as a novel class of targeted therapy active in breast, ovarian and pancreatic cancer with a deficiency in homologous repair (HR) DNA system [12,13]. The use of PARP inhibitors in the presence of a defective HR system stands on the so-called synthetic lethality: a single gene mutation is not lethal, but the simultaneous inactivation of more genes/proteins leads to cellular death [13]. BRCA1 and BRCA2 proteins play a key role in HR system, and their alterations increased susceptibility to drugs that induce double strand breaks in DNA, such as platinum-based chemotherapies. In *BRCA1/2* defective tumors, PARP pathway usually detects DNA damage and promotes its repair, so its inhibition favors the accumulation of DNA damage responsible for the death of tumor cells [14]. 

Different PARP inhibitors have been evaluated, among these veliparib was tested in 16 previously treated PDAC patients harboring *BRCA1/2* or partner and localizer of BRCA2 *(PALB2)* mutations; no confirmed response was observed, but one patient showed a partial response and four a stable disease [15]. Another PARP inhibitor is rucaparib that was tested after one or two prior chemotherapy regimens in a phase II trial, 19 PDAC *BRCA1/2* mutated patients were enrolled: two achieved a complete response and two a partial response. Interestingly, three out of the four responders harbored somatic *BRCA1/2* mutations [16]. 

Recently, the phase III study Pancreatic Cancer Olaparib Ongoing (POLO) evaluated the role of maintenance olaparib, a PARP inhibitor, in patients with *BRCA1* and *BRCA2* germline pathogenic mutations who were not progressed after 16 weeks of first-line platinum based chemotherapy, reporting an improved progression free survival (PFS) in comparison to the placebo group, while no benefit in overall survival (OS) was observed [17]. On the basis of this study the Food and Drug Administration (FDA) in 2019 approved olaparib in PDAC with known pathogenic *BRCA1/2* germline mutations [9].

In this context, the early assessment of *BRCA1* and *BRCA2* mutational status is crucial to identify patients who can achieve a better response with platinum-based chemotherapy as first-line regimen and who can be subsequently treated with PARP inhibitors. Independent of familial history, germline or somatic mutations in *BRCA1* and *BRCA2* genes occur in 5–10% [18] of patients with pancreatic cancers, with a prevalence for *BRCA2* ranging from 3.6–7% and <3% for *BRCA1* [19]. 

Although the latest guidelines recommended germline *BRCA1/2* testing to select PDAC patients eligible for olaparib maintenance treatment, *BRCA1* and *BRCA2* somatic mutations have been reported in 2% and 7–9% of unselected PDAC patients, respectively [19]. Moreover, Mohyuddin and collaborators in a recent meta-analysis found that outcomes for patients with somatic *BRCA1/2* mutations treated with PARP inhibitors appear similar to those of patients with germline alterations [20], thus suggesting that somatic mutations should be evaluated as well as germline ones. In this scenario, negative germline reports may advise a tumor study for the determination of somatic variants [21] and the possibility to perform *BRCA1/2* testing on tumor tissues would allow us to detect both germline and somatic alterations. 

However, to perform molecular analyses on pancreatic tumor tissues can be challenging, mainly because of the paucity of biological material from traditional fine needle aspirations (FNA) or endoscopic ultrasound (EUS) biopsies in advanced cases [22] and only few data are available about technical aspects related to *BRCA1/2* testing on pancreatic formalin-fixed and paraffin-embedded (FFPE) specimens [23].

Herein, we aim to specifically evaluate the feasibility of *BRCA1/2* testing by using an amplicon based Next Generation Sequencing (NGS) panel on a consecutive series of FFPE pancreatic tumor specimens, including FNAs, biopsies and surgical resections (tissue standard sections and macrosections). In particular, we focused on sample adequacy, failure rate and mutation rate of *BRCA1/2* tissue testing, pointing out some pre-analytical and analytical parameters impacting on the molecular analysis, such as formalin fixation, tumor representativeness and DNA fragmentation and concentration. 

## 2. Materials and Methods

### 2.1. Study Population

In this study, we evaluated for *BRCA1/2* testing thirty-seven clinical FFPE tumor specimens consecutively collected at the Unit of Pathological Anatomy—University Hospital of Pisa, from January 2020 to December 2020. Samples from primary and metastatic tumor sites included biopsies, cell-blocks from FNA and surgical specimens (Table 1). 

Sampling of fine-needle biopsies (FNB) was performed by 1 or 2 passes using a 18 gauge (G) needle by an EUS tissue acquisition (TA). FNA specimens were obtained by EUS using 10-mL syringe suction in a single pass of 22 G in two cases, 18 G and 21 G in one case for each.

For all cases, a confirmed histological and clinical diagnosis of pancreatic adenocarcinoma was available.

This study was conducted conforming to the principles of the Declaration of Helsinki of 1975. All cases were completely anonymous, and no sensitive data were used.

### 2.2. BRCA1 and BRCA2 Test

Serial 10-μm sections were obtained from FFPE tumor tissues, and the last section was stained with hematoxilin-eosin (H&E), the tumor area was marked, and the percentage of tumor cells was estimated by expert pathologists (CD and GF). The tumor tissue was manually macrodissected from one to three unstained sections after a xylene/ethanol based deparaffinization protocol. DNA was purified using the spin column procedure by the QIAamp DNA Mini Kit (Qiagen, Hilden, Germany) according to the manufacturer’s instructions, and finally reconstituted in 40 μL of elution buffer. 

*BRCA1*/2 testing was performed using the amplicon based NGS panel Myriapod NGS-LT BRCA 1–2 (Diatech Pharmacogenetics, Jesi, Italy), which allows us to sequence all exons and exon-intron boundaries of *BRCA1* and *BRCA2* genes.

According to the manufacturers, purified DNA underwent quantitative and qualitative evaluation by a duplex fluorescent-based qPCR assay consisting of two primer probe oligos sets that amplify two highly conserved genomic regions, whose amplification ratio was used to evaluate DNA fragmentation. A human genomic DNA (50 ng/μL) was serially diluted to obtain a qPCR standard curve ranging from 50 to 0.005 ng/µL in order to quantify DNA from clinical samples. Sequencing libraries were prepared from 5 to 25 nanograms of total DNA, and each library was then identified by a unique IonXpress barcode (Thermo Fisher Scientific, Waltham, MA, USA), enriched and purified following the used NGS panel protocol. The quantity of DNA libraries was assessed by the Qubit^®^ 4.0 fluorometer (Thermo Fisher Scientific) and diluted to obtain 100 µL of pooled libraries concentrated at 8 pM for the clonal amplification by emulsion PCR. Emulsion PCR and template-positive Ion Sphere Particles (ISPs) enrichment were performed manually according to Ion 520^TM^ & Ion 530^TM^ Kit–OT2 kit (Thermo Fisher Scientific). Sequencing reactions were performed on an Ion 530 chip and run on the Ion S5 Sequencing System (Thermo Fisher Scientific). Sequencing data were analyzed using the Torrent Suite™ Software, and variant calling performed by the Myriapod^®^ NGS Data Analysis Software (Diatech Pharmacogenetics). 

Minimum accepted coverage was 500×, allowing a variant limit of detection of 5%. Variants were reported according to the Human Genome Variant Nomenclature and classified on the basis of indications by the American College of Medical Genetics and Genomics/Association for Molecular Pathology [24] and the ENIGMA (Evidence-based Network for the Interpretation of Germline Mutant Alleles) guidelines (http://www.enigmaconsortium.org, accessed on 2 April 2021). Pathogenic and likely pathogenic mutations were regarded as deleterious mutations and predictive of response to PARP inhibitors, while variant of unknown significance (VUS) was also annotated.

## 3. Results

The main results are reported in Table 1. Briefly, 37 consecutive clinical cases were collected from 13 females and 24 males with pancreatic adenocarcinoma. *BRCA1/2* testing was performed on 32 out of 37 FFPE samples. 

In detail, twenty surgical samples were analyzed, all of them had a percentage of tumor cells superior to 20%. Six cases failed the NGS analysis, they were all macrosections presenting a low DNA concentration and a high DNA fragmentation level. Only one sample with a good DNA concentration [8 ng/µL] and a low fragmentation failed the test.

Thirteen biopsies were included in our study: three biopsies (PDAC liver metastasis) had a percentage of tumor cells inferior to 10% and so below the limit of detection of the test (5%), ten cases successfully underwent NGS analysis, including samples with a suboptimal DNA concentration. All the analyzed biopsies presented a low/medium DNA fragmentation level.

Only two cell-blocks from FNAs were analyzed by NGS providing good results, two cases were not adequate because of a low percentage of tumor cells. Inadequate cases were sampled one by a 21 G and one by a 22 G needle.

Overall, in twenty-six cases, the NGS test gave valuable results, with three cases (11.5%) harboring pathogenic mutations in *BRCA2* gene, and one case with a VUS within the *BRCA1* gene (3.8%) (Table 1). All mutations had a variant allele frequency (VAF) compatible with a heterozygous status and were confirmed as germlines. 

## 4. Discussion

Pancreatic cancer, particularly PDAC, has a poor prognosis and limited treatment options [3]. Although comprehensive genomic analyses have reported a high rate of PDAC patients harboring actionable alterations [25], combined chemotherapy regimens based on FOLFIRINOX or gemcitabine with nab-paclitaxel are still the standards of care for the treatment of locally advanced and metastatic diseases. Development and approval of targeted therapies in this setting of patients have always been hampered by the great tumor heterogeneity and by the paucity of biological material usually available for locally advanced and metastatic stages (80% of cases) [3,9].

EUS-TA is the primary method for obtaining tissue samples from pancreatic cancer and has the potential to provide tumor DNA for NGS analysis as reported in different studies [26,27,28,29]. In particular, it has been demonstrated that the adequacy of EUS-TA samples for NGS ranged from 60% to 100% [26,30,31,32]. At present, different sizes of needles for EUS-TA are available: 18- to 25-gauge needles. Theoretically, larger needles can obtain a larger amount of material. However, in a recent network meta-analysis, needle type (FNA vs. FNB) or gauge (19 G vs. 22 G vs. 25 G) were compared, and no specific EUS-guided tissue sampling technique proved to be superior with regard to diagnostic accuracy and sample adequacy [33,34].

Olaparib, a PARP inhibitor, has recently been approved as maintenance therapy for PDAC patients harboring a germline *BRCA1* or *BRCA2* pathogenic mutation, who have received first-line platinum-based chemotherapy [11,17]. In this context, the assessment of *BRCA1/2* mutational status is required to select patients to treat with a platinum-based chemotherapy as first-line regimen and who can then benefit from PARP inhibitors. 

Currently, *BRCA1/2* germline testing is recommended for all PDAC patients independently of their familial history. However, PDAC patients with somatic *BRCA1/2* mutations can also respond to PARP inhibitors [16,20]. In this context, *BRCA1/2* analysis on tumor tissues would allow us to detect both germline and somatic alterations and has already become the standard procedure in other cancer types, such as ovarian carcinoma [35]. 

Although the NGS system allows us to perform extensive molecular analysis on a limited nucleic acid input, the molecular profiling of pancreatic tumor tissues is not yet a part of clinical practice and only few data are available about its feasibility and particularly about *BRCA1/2* testing on this type of specimens [22,25]. In 2019, Okuwaki and collaborators evaluated the adequacy of a series of 20 FFPE pancreatic samples obtained by EUS fine-needle aspiration biopsy to evaluate the BRCAness status by multiplex ligation-dependent probe amplification (MLPA), reporting a success rate equal to 75% with one positive case [23]. MLPA is a valuable technique to evaluate large gene deletions and amplifications, which constitute about 12% of all *BRCA1/2* pathogenic variants, and it can be useful in some cases to complete and confirm NGS analyses [36].

Herein, we evaluated for *BRCA1/2* testing 37 consecutive PDAC clinical specimens in order to identify the most important technical aspects impacting on the outcome of molecular analysis. In particular, this is a feasibility study investigating the adequacy of different types of pancreatic FFPE specimens for an NGS amplicon-based test for the analysis of *BRCA1/2* mutational status. We reported failure and success rates in relation to sample type and relevant pre-analytical and analytical factors, such as sample processing (i.e., formalin fixation), representativeness of the tumor, nucleic acid concentration and fragmentation. Thirty two out of 37 cases (86.5%) had an adequate percentage of tumor cells for the test (>10%), while five cases were excluded (13.5%). Among the few available data, Pishvain et al., by collecting 640 PDAC tumor biopsies, mainly from metastatic sites, found that up to 99% were adequate for NGS analysis and 96% provided valuable results [25]. In our study, a low percentage of tumor cells was reported only in two out of 13 biopsies, most of which were from liver metastatic sites. Young and collaborators have demonstrated on a retrospective and non-consecutive series that cell-blocks from pancreatic FNA samples can be adequate for NGS test with a success rate of 100% (23/23 analyzed cases) [26], whereas in our consecutive series, two out of four FNAs had an insufficient percentage of tumor cells. Further data on pancreatic FNA specimens are needed to evaluate the adequacy rate for NGS testing of tumor samples obtained using this technique, crucial for the management of PDAC patients.

The use of an amplicon based method for *BRCA1/2* testing, which usually requires less DNA input in comparison to hybrid capture strategy [21], allowed us to also analyze samples with a suboptimal DNA yield. Good NGS quality metrics were reported for 26 out of 32 specimens with a success rate of 81.2%.

Notably, all samples that failed NGS analysis were FFPE large format sections or macrosections. Tissue macrosections have the advantage to allow the histological evaluation of a large part of the organ or site of interest [37], but pre-analytical factors and particularly fixation procedures may impact on DNA quantity and quality more than standard tissue sections. Macrosections, because of their size, can undergo over-fixation and this can increase DNA and protein degradation [38]. Indeed, we found that most of tissue macrosections included in this study (nine out of fourteen) presented a highly fragmented and low concentrated DNA as evaluated by qPCR. On the other hand, biopsies and cell-blocks had an overall low fragmented DNA, and standard tissue sections from surgical resections had a medium DNA fragmentation level. This confirms how crucial it is for the molecular pathology laboratory to carefully check for pre-analytical conditions and particularly for fixation type and time [21].

For 3 out of 26 cases, a *BRCA2* germline pathogenic mutation was found, with a mutation rate (11.5%) slightly higher than reported in the literature, likely due to the low number of analyzed cases. It has to be underlined that both on blood and on tumor tissue *BRCA1/2* testing should be cautionary performed, considering that about 10% of PDAC have a genetic cause and a genetic counselling is mandatory to correctly treat all the *BRCA1/2* germline mutation carriers.

Besides the identification of *BRCA1/2* pathogenic variants, the analysis of tumor tissues can have other advantages. Particularly, it could lead to identify primary or acquired resistance mechanisms to platinum-based chemotherapy or PARP inhibitors, such as accumulation of somatic mutations restoring the HR system on cancer cells and to evaluate allelic specific loss of heterozygosity [9,16,39]. Additionally, in 2018, Pishvaian and collaborators found a high rate of actionable genomic and proteomic alterations in PDAC, mainly affecting DNA repair genes, but also other therapeutic targets [25]. In this context, *BRCA1/2* testing on tumor tissues could be combined with other biomarker evaluations, thus saving time and biological materials and allowing us to identify a higher number of patients eligible for available targeted therapies or to be enrolled in ongoing clinical trials. In the last years, cell-free tumor DNA (ctDNA) has been acquiring a great relevance for precision medicine, since it allows us to perform a molecular characterization of the tumor in a less invasive manner and to overcome issues related to tumor heterogeneity. Although few data are available, the analysis of ctDNA could provide several advantages in clinical practice of PDAC [40] and it should be useful also for the evaluation of *BRCA1/2* gene mutations, particularly when tumor tissue is not available or adequate. Moreover, interesting data have been published about the possibility to evaluate on ctDNA also *BRCA1/2* reversion mutations to predict primary and acquired resistance to PARP inhibitors [41].

This study has some important limitations mainly related to the low number of analyzed cases and particularly FNAs and biopsies by EUS that have a crucial role in clinical practice. Moreover, our analysis focused only on *BRCA1* and *BRCA2* testing and it could be useful to evaluate also more comprehensive NGS panel including other genes involved in DNA HR system. 

In conclusion, only few data are available about *BRCA1/2* molecular testing on clinical FFPE specimens from pancreatic tumors. Herein we provided evidence about the feasibility of this testing also on small biopsies and from limited amounts of input DNA. However, it is necessary to collect more data to better understand how to prioritize and select pancreatic samples for molecular analysis, thus optimizing pre-analytical and analytical protocols; and to evaluate role and cost-effectiveness of *BRCA1/2* tissue testing in the diagnostic algorithm of PDAC.

## Figures and Tables

**Table 1:** *BRCA1/2* testing results.

ID	Sample Characteristics					DNA		NGS Test		
	Procedure	Site	Type	Diagnosis	% Tumor Cells	Concentration ng/µL	Fragmentation Level	Coverage	*BRCA1*	*BRCA2*
1	Surgical resection	Pancreas	Macrosection	PDAC	40%	3.5	High	2668	WT	WT
2		Pancreas	Standard section	PDAC	80%	27.43	Medium	3352	WT	WT
3		Pancreas	Standard section	PDAC	60%	1.22	Medium	2687	WT	WT
4		Pancreas	Macrosection	PDAC	40%	1.57	High	<500	Failed	Failed
5		Pancreas	Macrosection	PDAC	30%	0.52	High	<500	Failed	Failed
6		Pancreas	Macrosection	PDAC	40%	1.25	Medium	8333	WT	Exon 14: c.7180A > T; p.(Arg2394 *) VAF: 51.8%
7		Pancreas	Macrosection	PDAC	40%	0.51	High	<500	Failed	Failed
8		Pancreas	Macrosection	PDAC	50%	2.15	High	5388	WT	WT
9		Pancreas	Macrosection	PDAC	50%	2.0	Medium	4461	WT	WT
10		Pancreas	Macrosection	PDAC	40%	0.77	High	<500	Failed	Failed
11		Pancreas	Standard section	PDAC	60%	2.27	High	3588	WT	WT
12		Pancreas	Macrosection	PDAC	30%	7.49	High	2726	WT	WT
13		Pancreas	Macrosection	PDAC	70%	0.48	High	<500	Failed	Failed
14		Pancreas	Macrosection	PDAC	50%	2.45	Medium	5553	WT	WT
15		Liver	Standard section	Metastasis of PDAC	50%	4.63	Medium	4062	WT	WT
16		Pancreas	Standard section	PDAC	20%	0.7	High	3141	WT	WT
17		Pancreas	Macrosection	PDAC	80%	4.24	High	3419	WT	WT
18		Pancreas	Macrosection	PDAC	60%	0.49	Medium	5721	WT	WT
19		Pancreas	Standard section	PDAC/IPMN	30%	0.76	Medium	6691	WT	WT
20		Pancreas	Macrosection	PDAC	60%	8.18	Low	<500	Failed	Failed
21	Tissue biopsy	Liver	Standard section	Metastasis of PDAC	50%	2.66	Low	3564	WT	WT
22		Liver	Standard section	Infiltration of PDAC	80%	7.88	Low	3479	WT	WT
23		Liver	Standard section	Metastasis of PDAC	50%	0.97	Low	3050	WT	Exon 11: c.6346delC; p.(His2116fs) VAF: 51.3%
24		Pancreas	Standard section	Infiltration of PDAC	20%	2.04	Medium	3582	WT	WT
25		Liver	Standard section	Metastasis of PDAC	<10%	/	/	/	Not performed	Not performed
26		Liver	Standard section	Metastasis of PDAC	50%	0.87	Low	4433	WT	WT
27		Liver	Standard section	Localization of ADC	40%	0.97	Low	2743	WT	WT
28		Liver	Standard section	Localization of ADC	50%	0.42	Low	13617	WT	WT
29		Liver	Standard section	Localization of ADC	<10%	/	/	/	Not performed	Not performed
30		Liver	Standard section	Metastasis of PDAC	60%	1.12	Low	4552	WT	WT
31		Liver	Standard section	Metastasis of PDAC	50%	2.66	Low	3564	WT	WT
32		Liver	Standard section	Metastasis of PDAC	70%	0.8	Low	2947	WT	Exon 24: c.9154C > T; p.(Arg3052Trp) VAF: 59.9%
33		Liver	Standard section	Localization of ADC	<10%	/	/	/	Not performed	Not performed
34	FNA	Pancreas	Cell-block	ADC	10%	3.29	Low	3690	Exon 11: c.2077G > A; p.(Asp693Asn) VAF: 33.7%	WT
35		Pancreas	Cell-block	ADC	<10%	/	/	/	Not performed	Not performed
36		Pancreas	Cell-block	ADC	50%	1.69	Low	3519	WT	WT
37		Pancreas	Cell-block	ADC	<10%	/	/	/	Not performed	Not performed

For each case, NGS results are reported in relation to sample characteristics (histological diagnosis, sample type and percentage of tumor cells) and DNA quantity and quality evaluated by qPCR. The percentage of tumor cells was independently determined by two expert pathologists, and minimum accepted coverage for NGS analysis was 500x. Abbreviations: FNA, fine-needle aspiration; PDAC, pancreatic ductal adenocarcinoma; ADC, adenocarcinoma; VAF, variant allele frequency; WT, wild type.

## Data Availability

Data available from the corresponding author on request.
